# targeting gamma‐secretase in the Dominantly Inherited AD patients

**DOI:** 10.1002/alz.090508

**Published:** 2025-01-09

**Authors:** Eric McDade

**Affiliations:** ^1^ Washington University in St. Louis, School of Medicine, St. Louis, MO, USA; Washington University School of Medicine, St. Louis, MO, USA; Knight Alzheimer Disease Research Center, St. Louis, MO USA

## Abstract

Although amyloid b immunotherapies offer great potential for prevention or delay of symptoms in dominantly inherited AD (DIAD), the mechanism of action of this class of medications does not address the underlying mechanism of most DIAD mutations. Moreover, the need for repeated IV infusions or sub‐cutaneous injections with Ab immunotherapies may prove challenging for long‐term prevention approaches. The majority of DIAD mutations appear to affect the interaction of the gamma‐secretase enzyme with the Amyloid Precursor Protein (APP) making this enzyme an attractive target for disease modification. However, emerging evidence also suggests that different DIAD mutations can have significantly different effects on the processing of APP, which suggests that targeting this enzyme may have mutation dependent effects as well.

Developments in the understanding of the timing of amyloid b‐dependent changes in DIAD and the development of accurate measurements of Ab now enable precision‐based approaches necessary for targeting gamma‐secretase. Moreover, the presence of clinical trial platforms for DIAD facilitate the implementation of trials for targeting gamma‐secretase.

In this session, we will review approaches to testing next generation gamma‐secretase therapies in DIAD. Specifically, we will outline prevention approaches in DIAD that leverage fluid‐based biomarker measurements of Ab ‐peptides and sensitive, brief cognitive assessments to determine optimal mutation specific dosing approaches‐ i.e. precision medicine approach. Combining plasma, CSF and molecular PET imaging, we will outline how to assess gamma‐secretase targeting therapies for different mutations in DIAD along a timescale that utilizes early pharmacodynamic effects on Ab ‐peptides to predict long‐term effects on Ab ‐and downstream biomarkers of AD.

